# Co-Occurrence of Avoidant/Restrictive Food Intake Disorder (ARFID) and Schizophrenia-Spectrum Disorders: A Comprehensive Review

**DOI:** 10.3390/jcm15051704

**Published:** 2026-02-24

**Authors:** Maria Benedetta Anesini, Stella Margoni, Lorenzo Moccia, Sara Barbonetti, Luca Onori, Elena Lucia Valle, Antonio Maria D’Onofrio, Francesca Focà, Mario Pinto, Georgios D. Kotzalidis, Fabio Conti, Gabriele Sani

**Affiliations:** 1 Department of Neurosciences, Section of Psychiatry, Università Cattolica del Sacro Cuore, 00168 Rome, Italy; mbenedetta@hotmail.it (M.B.A.); stella.margoni98@gmail.com (S.M.); sara.barbonetti@gmail.com (S.B.); luca.onori@yahoo.it (L.O.); elenalucia.v@gmail.com (E.L.V.); mario.pinto@guest.policlinicogemelli.it (M.P.); giorgio.kotzalidis@gmail.com (G.D.K.); gabriele.sani@unicatt.it (G.S.); 2 Department of Neurosciences, Head-Neck and Chest, Section of Psychiatry, Fondazione Policlinico Universitario Agostino Gemelli IRCCS, 00168 Rome, Italy; 3 Unit of Clinical Psychology, Fondazione Policlinico Universitario Agostino Gemelli IRCCS, Università Cattolica del Sacro Cuore, 00168 Rome, Italy; francesca.foca@guest.policlinicogemelli.it; 4 Neomesia Kos Group, Institute of Neurosciences, Via Nomentana 1362, 00137 Rome, Italy; fabio.conti@neomesia.com

**Keywords:** avoidant/restrictive food intake disorder, ARFID, psychosis, schizophrenia, avoidance, delusions

## Abstract

**Background/Objectives**: Avoidant/restrictive food intake disorder (ARFID) and psychotic disorders are clinically distinct conditions yet occasionally co-occur in ways that complicate assessment and treatment. ARFID is characterised by avoidance of food due to sensory sensitivities, fear of aversive consequences, or low interest in eating, without body-image distortion. Recent meta-analytic evidence suggests that ARFID affects a substantial proportion of the population and is associated with a considerable social burden. Psychosis is characterised by positive symptoms (hallucinations and delusions), negative symptoms (avolition, blunted affect, and social withdrawal), and cognitive impairments affecting thought, perception, and behaviour. **Methods**: Across the limited literature, shared mechanisms between ARFID and psychotic disorders appear to converge on pathological avoidance, which may arise from sensory overstimulation, obsessive–compulsive features, or delusional beliefs about food. Case reports indicate that psychosis may both mimic ARFID and exacerbate food avoidance, while severe malnutrition can itself precipitate or worsen psychotic symptoms, blurring diagnostic boundaries. **Results**: Abnormalities in interoception, sensory sensitivity, and disrupted perception of bodily signals are manifestations of both ARFID and psychosis, suggesting a potential bridging pathway contributing to vulnerability and clinical overlap. **Conclusions**: Given the paucity of empirical studies and the reliance on isolated case reports, systematic investigation is mandatory and necessary to clarify shared mechanisms, refine differential diagnosis, and guide integrated treatment approaches. Given the heterogeneity of symptoms in comorbid patients, a personalised approach is suggested for treating these patients.

## 1. Introduction

Avoidant/restrictive food intake disorder (ARFID) is characterised by a persistently inadequate nutritional intake that cannot be accounted for by concerns about weight or body shape. Individuals typically restrict food because of marked sensory sensitivities, low appetite or interest in eating, or fear of adverse consequences such as choking or vomiting. These patterns can lead to significant medical impairment, the need to rely on nutritional supplementation, and remarkable psychosocial impairment [1]. Although initially thought to be relatively uncommon, recent research indicates that ARFID is more prevalent than previously suggested, particularly among children and adolescents, and increasingly recognised in adults as well [2]. Epidemiological and clinical studies have identified a wide range of ARFID presentations, from highly selective eating to pronounced sensory-based avoidance, reflecting a heterogeneous condition with multifactorial origins—including temperamental predispositions, neurobiological vulnerabilities, and environmental experiences [2].

Emerging genetic research indicates that ARFID-related traits have a heritable component distinct from that of traditional eating disorders, suggesting that ARFID represents a separate psychopathological construct with its own aetiological basis [3,4].

Clinically, children and adolescents with ARFID frequently exhibit medical consequences of undernutrition and marked psychological distress, despite the absence of weight- or shape-related concerns. Evidence also suggests that ARFID may persist into adulthood, with its core features remaining identifiable across developmental stages [5]. This heterogeneous and developmentally fluid profile is particularly relevant when considering the potential intersections between ARFID and psychotic phenomena, especially given the role of neurodevelopmental factors in both conditions [6,7,8].

Differentiating ARFID from other eating disorders is essential, as the mechanisms underlying food restriction differ markedly. Comparative studies show that individuals with ARFID tend to exhibit heightened harm avoidance, sensory sensitivity, and avoidance-oriented traits. Personality research further indicates that ARFID is more often characterised by emotional reactivity and sensory vulnerability, contrasting with the cognitive and motivational patterns typically associated with other restrictive eating presentations [9].

Psychopathologically, although ARFID shares some vulnerabilities with traditional eating disorders, its symptom structure and comorbidity patterns differ [9]. For example, depressive and obsessive-compulsive symptoms are more characteristic of anorexia, whereas ARFID shows stronger associations with anxiety, somatic fears, and neurodevelopmental features [9,10,11].

Cognitive studies also support this diagnostic separation, in that individuals with ARFID do not typically exhibit the distorted weight- and shape-related cognitions seen in anorexia [12,13], but instead show apprehension linked to sensory overload, somatic risk, or anticipated harm from eating [9]. This distinction remains observable across developmental stages, suggesting that restrictive eating in ARFID is underpinned by fundamentally different motivational, perceptual, and possibly biological processes [14].

Such findings provide an important basis for examining overlaps between ARFID and psychotic presentations, particularly where sensory abnormalities, threat-based interpretations, or belief rigidity come into play.

Current conceptualisations situate psychosis on a continuum ranging from subclinical experiences to persistent disorders, shaped by neurodevelopmental trajectories and environmental exposures. The persistence and impairment of psychotic symptoms appear linked to neurodevelopmental deviations, including atypical sensory processing, cognitive inflexibility, and altered stress reactivity [15]. Genetically informed research further supports the interplay between neurodevelopment and psychosis, demonstrating shared heritable factors across synaesthesia, autistic traits, and psychiatric vulnerability—highlighting how atypical sensory-perceptual processing may constitute a common risk dimension [16]. Neurobiological accounts also emphasise the contribution of astrocytic dysfunction within broader neurodevelopmental frameworks of schizophrenia, illustrating how cellular-level alterations may intersect with perceptual and cognitive disturbances [17].

This body of work provides a conceptual bridge to ARFID, where sensory hypersensitivity, early neurodevelopmental difficulties, and fear-driven avoidance may encompass domains also implicated in psychosis-proneness [18].

Restrictive eating behaviours and eating disorders are reported in a subset of individuals with psychosis, where such comorbidity may negatively influence illness progression [15,19]. Evidence from high-risk populations further indicates that disordered eating can precede the onset of psychosis and is linked to poorer functional outcomes [20]. The motivational drivers of food restriction in psychosis can be diverse, encompassing persecutory fears (e.g., food contamination or poisoning), sensory disturbances, and anomalous bodily experiences. Some of these factors partially overlap with ARFID presentations, particularly where avoidance is rooted in perceived threat, heightened sensory reactivity, or distorted interpretations of somatic cues [21].

Although this parallelism does not imply that ARFID and psychosis share some common unified mechanism, it points to potential zones of convergence that warrant systematic exploration.

Given the paucity of research on co-occurring ARFID and psychosis, accurate differential diagnosis is crucial to avoid misattributing food avoidance and compromising care. Potential points of overlap, such as sensory hypersensitivity, threat-based interpretations, neurodevelopmental vulnerabilities, and rigid cognitive styles, suggest a plausible, though largely unexamined, interface between the two conditions. The evidence base remains very limited, highlighting the need for studies on shared mechanisms and clinical outcomes. On this basis, we opted to conduct a narrative review on the coexistence between ARFID and the psychotic spectrum. This narrative review therefore aims to synthesise and critically evaluate the literature available to date on this understudied area.

## 2. Materials and Methods

This work was conceived as a narrative review aimed at exploring the clinical and conceptual interface between ARFID and schizophrenia-spectrum disorders. Given the emerging nature of this topic and the limited number of available empirical studies, a flexible and exploratory approach was adopted rather than a systematic or exhaustive review methodology.

A literature search was conducted on 14 February 2026 using the PubMed, Scopus, and PsycInfo databases to identify relevant publications addressing the co-occurrence or phenomenological overlap between ARFID and psychosis. Search terms included combinations of “ARFID” or “avoidant/restrictive food intake disorder” with terms referring to psychotic and psychosis-adjacent conditions (e.g., psychosis, psychotic, schizophrenia, schizoaffective, bipolar, mania, schizotypal, paranoid). The detailed search strategy for each of the investigated databases is shown in the Supplementary Material, Supplementary Table S1. The purpose of this search strategy was to capture a broad range of clinically and theoretically relevant contributions rather than to ensure comprehensive coverage. We included studies dealing with ARFID and presenting data on psychotic symptoms or conditions since the inception of the investigated databases up to the date we conducted the search with no language exclusion; original articles and case reports or series were considered.

Given the scarcity of research in this area, case reports and brief clinical communications were considered eligible for citation when they provided detailed descriptions of restrictive eating patterns consistent with ARFID in the context of psychotic or psychosis-spectrum presentations. In contrast, review articles, meta-analyses, guidelines, and consensus papers were not included, as the focus of the present work was on primary clinical observations and original empirical contributions. The risk of bias of the articles considered here is shown in the Supplementary Table S2. However, we hand-searched the reference lists of reviews to identify possible eligible studies.

Although there were no time or language restrictions in our searches, the retrieved articles were all published in English. The selection of studies was guided by their relevance to key themes of interest, including diagnostic challenges, phenomenological overlap, hypothesised mechanisms, and clinical implications at the intersection of ARFID and psychosis. Data were synthesised qualitatively to highlight patterns, points of convergence, and areas of uncertainty within the existing literature, rather than to derive quantitative estimates or causal inferences.

## 3. Results

Our search, carried out on 14 February 2026, produced 14 records on PubMed, 11 on Scopus and five on PsycInfo/PsycArticles for a total of 30 records, of which nine were duplicates, bringing the grand total to 21 records (Supplementary Table S1). Of these, seven did not refer to psychosis, six were case reports, three were epidemiological, three were on the psychometrics of ARFID rating scales, one was a survey, and one was an opinion. With respect to PubMed, Scopus produced seven new articles and four duplicates, while with respect to PubMed and Scopus, APA PsycInfo/PsycArticles produced no new records; all five records it produced were duplicates.

The available literature examining the co-occurrence of ARFID and schizophrenia-spectrum disorders is sparse, patchy and heterogeneous. The evidence base consists of a small number of observational studies and isolated clinical case reports, reflecting the early stage of investigation in this area.

One cohort study investigated children and adolescents with clinical or subclinical ARFID [2]. This study examined a sample from both treatment-seeking and community settings and assessed psychopathology using structured diagnostic interviews. Although individuals with a current or lifetime diagnosis of psychotic disorders were explicitly excluded, the findings remain informative in delineating the broader psychiatric vulnerability associated with ARFID. The authors reported a high burden of psychiatric comorbidity, including anxiety disorders, obsessive–compulsive symptoms, depressive disorders, bipolar-related features, and neurodevelopmental conditions. This pattern suggests substantial transdiagnostic overlap along dimensions such as avoidance, sensory sensitivity, affective dysregulation, and cognitive rigidity. While psychotic disorders could not be directly assessed within this cohort, the clustering of these vulnerabilities supports the plausibility of shared risk pathways linking ARFID to psychosis-spectrum presentations [2]. However, the epidemiology and comorbidity of ARFID cannot be investigated only by studies investigating ARFID-only populations, as these cannot assess its impact in the general population. Studies using clinical diagnoses with clinical records and interviews report prevalences around 4–5% for quality effects and 11% for random effects [22], while those investigating the issue with the use of surveys via telephone or online generally report higher rates. A meta-analysis indicated figures of 0.3% to 15.5% in non-clinical samples, while in services dedicated to eating disorders it was 5–22.5%; however, in specialist feeding clinics the prevalence rates ranged from 32% to 64%, which is quite a high rate matching the figure obtained in surveys [23]. The prevalence rates of ARFID in various populations are shown in Table 1.

Complementary evidence was provided by a clinical survey investigating psychiatric comorbidity in individuals with ARFID. This study similarly highlighted marked heterogeneity in clinical profiles and a high prevalence of anxiety-related and neurodevelopmental conditions. Although psychotic disorders were uncommon, avoidance-based eating behaviour emerged as a central and transdiagnostic feature, potentially extending across a range of severe psychiatric conditions [43]. Together, these observational findings suggest that ARFID is embedded within a broader landscape of psychiatric vulnerability rather than representing an isolated eating disorder phenotype.

More direct evidence of an association between ARFID and psychosis derives from a small number of clinical case reports, which describe markedly diverse presentations across age groups and diagnostic contexts.

One report described a 32-year-old man with chronic schizophrenia and comorbid obsessive–compulsive disorder who developed severe food restriction consistent with ARFID. The case illustrated the diagnostic complexity of distinguishing ARFID-related avoidance from psychosis-driven refusal of intake. Prominent sensory hypersensitivity, rigid food preferences, and progressive malnutrition were observed, ultimately resulting in a fatal medical outcome [48].

A second case involved a 27-year-old woman presenting with restrictive eating, psychotic symptoms, and severe metabolic alkalosis in the context of Gitelman syndrome. The authors emphasised a bidirectional interaction in which malnutrition and medical instability appeared to exacerbate psychotic symptoms, while psychosis itself intensified avoidance of food and fluids. This case underscored the difficulty of disentangling primary ARFID, psychosis-related food refusal, and medical factors influencing eating behaviour [49].

A third report described a 16-year-old boy with early-onset, treatment-resistant schizophrenia, intermittent catatonia, and severe refusal to eat. In this case, food avoidance was conceptualised as ARFID-like behaviour triggered by religious delusions. Nutritional rehabilitation and antipsychotic treatment were both required to achieve partial symptom improvement, although relapse was frequent due to poor treatment adherence [50].

A further report described the case of a 16-year-old boy with ARFID symptoms who was hospitalised for severe gastrointestinal symptoms [51]. The boy was fed at the hospital and recovered from ARFID, thereby allowing for psychosis to emerge clearly, eventually receiving a diagnosis of “comorbid psychosis”; it should be underlined that as the eating symptoms were improving, misophonia, paranoia and command hallucinations along with suicidal and homicidal impulses were growing [51]. Also, this patient had familiarity for schizophrenia, which should have oriented the clinicians. It is known that ARFID-style eating can trigger psychotic-like symptoms, but these symptoms generally subside with eating symptom improvement, while in this case, we may consider the eating symptoms as triggered by the underlying psychosis.

**Figure 1:**
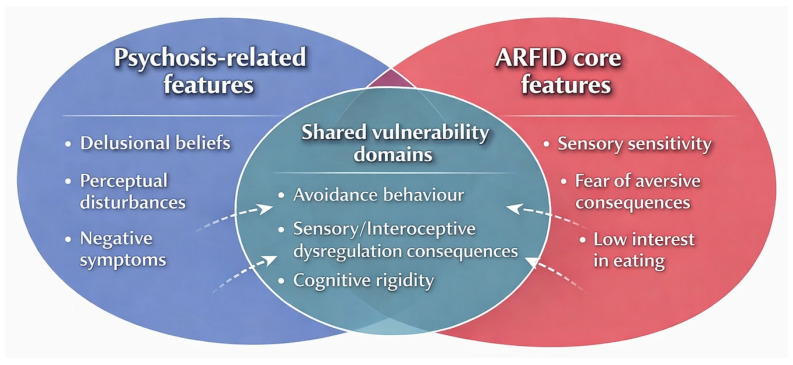
Hypothesised domains of overlap between ARFID-related restrictive eating behaviours and psychosis-spectrum features. The diagram illustrates shared transdiagnostic mechanisms at the intersection of psychotic phenomena and avoidant/restrictive food intake disorder (ARFID). While psychosis-related features include delusional beliefs, perceptual disturbances, and negative symptoms, and ARFID core features encompass sensory sensitivity, fear of aversive consequences, and low interest in eating, both conditions overlap on avoidance behaviour, sensory/interoceptive dysregulation, and cognitive rigidity, highlighting potential shared neurodevelopmental and psychopathological pathways.

Across the available studies, findings were highly heterogeneous, reflecting variability in age, psychiatric comorbidity, and the underlying reasons driving food avoidance. Psychosis appeared to intersect with ARFID-related presentations through multiple, mutually not exclusive pathways, including sensory hypersensitivity, avoidance driven by delusional content, malnutrition-induced cognitive and perceptual disturbances, and complex medical comorbidities reinforcing restrictive intake (Figure 1). Taken together, the limited and predominantly case-based nature of the evidence precludes us from drawing firm conclusions regarding the prevalence or the aetiology of ARFID. Nonetheless, the available data suggest that the interface between ARFID and psychosis is clinically meaningful, multifactorial, and currently underexplored, warranting further systematic investigation.

## 4. Discussion

Our searches for literature intersecting the domains of ARFID and psychosis produced heterogeneous studies pointing to the existence of ties between the two domains, but the fine details are currently missing. The question of which comes first is not resolved, but there are many pathways through which a psychosis may lead to ARFID-like behaviour. While distinct from picky eating, ARFID involves picky eating as one of its dimensions, at least with regard to the main screener used to identify it [52]. What emerges from the literature is that ARFID is a dangerous condition, possibly leading surreptitiously to malnutrition, and is not readily recognised.

### 4.1. ARFID as a Heterogeneous and Transdiagnostic Condition

ARFID is increasingly recognised as a highly impairing feeding and eating disorder with substantial medical, developmental, and psychosocial consequences. Epidemiological evidence suggests that its prevalence is higher than initially estimated, with a significant impact on child and adolescent functioning and healthcare utilisation [2,41,43,45]. Large-scale cohort studies indicate that ARFID spans a broad spectrum of severity and is embedded within a wider landscape of psychiatric vulnerability rather than representing an isolated eating disorder phenotype [39,42].

A defining characteristic of ARFID is its phenotypical heterogeneity. Sensory-driven avoidance, fear-based restriction, and low interest in eating may predominate at different developmental stages and fluctuate over time [40,50]. This variability distinguishes ARFID from other eating disorders, which are typically centred on weight and shape concerns. Consistent with this distinction, ARFID is associated with distinct motivational profiles, emotional features, and personality correlates, including avoidant, introverted, and cognitively rigid traits [5,9].

### 4.2. Psychiatric Comorbidity and Vulnerability Architecture

Comorbidity patterns in ARFID reveal frequent co-occurrence with anxiety disorders, obsessive–compulsive features, neurodevelopmental conditions, and, less commonly, psychotic symptoms [43]. Coexisting comorbidity is the rule rather than the exception [2,40]. Associations with suicidal ideation—particularly in the presence of dysmorphic concerns—have also been reported, alongside links with autistic traits and temperament profiles characterised by behavioural inhibition [53,54]. Together, these findings support the view of ARFID as a multidimensional condition at the intersection of affect regulation, sensory processing, and neurodevelopmental vulnerability.

Narcissistic traits have been described in a subset of individuals with ARFID, particularly those with rigid eating patterns and heightened sensitivity to bodily or interpersonal intrusion [55]. Disturbances in self-integration and interpersonal boundaries are also observed in schizophrenia-spectrum disorders, where they may manifest as defensive hypervigilance or narcissistic configurations [56,57]. Although these features arise within different diagnostic frameworks, both conditions may involve a fragile sense of self that increases vulnerability to dysregulated responses to somatic and affective stimuli.

### 4.3. Sensory and Interoceptive Processing as Points of Convergence

A key area of convergence between ARFID and psychosis involves abnormalities in sensory and interoceptive processing. Sensory hypersensitivity and heightened disgust responses to food textures are core features of ARFID across age groups [47]. Similarly, psychosis research has documented disruptions in the processing and integration of bodily signals, including altered interoceptive accuracy and predictive processing [58]. Disturbances in interoceptive signalling have also been implicated in altered self-experience and emotional dysregulation in psychosis [59].

These converging findings point to a transdiagnostic vulnerability characterised by atypical weighting of sensory input and impaired integration of bodily information. Importantly, this overlap should not be interpreted as evidence of shared diagnostic mechanisms, but rather as partially overlapping domains of dysfunction influencing symptom expression.

### 4.4. Clinical Overlap Between ARFID and Psychosis-Spectrum Presentations

Empirical evidence directly addressing the co-occurrence of ARFID and psychosis remains limited and heterogeneous. Observational studies document extensive psychiatric comorbidity and clustering of traits such as avoidance, sensory sensitivity, and affective dysregulation, which may interface with psychosis-spectrum vulnerability even in the absence of diagnosed psychotic disorders. In contrast, case reports [48,49,50] provide more direct illustrations of overlapping symptomatology, describing scenarios in which primary ARFID, delusion-driven food refusal, sensory hypersensitivity, and malnutrition-exacerbated psychotic symptoms interact in complex ways.

This imbalance between epidemiological data and case-based evidence highlights the difficulty of defining the boundary between ARFID and psychosis in clinical practice. Diagnostic challenges are particularly pronounced in adolescents and young adults, where developmental factors may blur distinctions between fear-based restrictive eating and psychosis-related food refusal.

Another question is that of the diachronic stability of ARFID. Few studies have observed the long-term outcomes of patients with ARFID, and only one examined its diagnostic stability [60]. One study based on retrospective ARFID diagnoses, which followed up patients for nearly 16 years, found all patients of a small sample (*n* = 19) with ARFID to endorse the same diagnosis, while having one current eating disorder or one other psychiatric disorder in 26.3% of cases each and no psychiatric diagnosis in 47.4% of cases; in contrast, patients originally diagnosed with anorexia nervosa showed a higher variability and diagnostic instability [61]. The only study published on the diagnostic stability of ARFID seen prospectively is the one by Kambanis et al. (2025) [60]; this study found substantial stability for ARFID, in that 44% of 100 patients aged 9–23 years with ARFID maintained their original diagnosis across both 1-year and 2-year follow-ups. Only 3% of the 100 ARFID patients transitioned to restrictive anorexia nervosa, while 12% achieved remission at both follow-ups. The picture was more confusing as to patients achieving remission and relapsing at the last follow-up (11%) and to those retaining the ARFID diagnosis at 1 year and remitting at 2 years (6%). All these data suggest that the whole picture is not yet clear, and further research is mandatory.

### 4.5. Screening for ARFID: Specific Tools Used to Detect ARFID

There are a few ARFID screeners that are routinely used in clinical practice. These were developed after ARFID became a psychiatric diagnosis in 2013 [62]. One is the Nine Item ARFID Scale (NIAS) [52], a 9-item scale. Each item is rated on a 0–6 Likert severity scale and yields scores on three dimensions, i.e., picky eating, low appetite and fear of aversive consequences of food. The tool has shown favourable psychometric properties, with high internal consistency, test–retest reliability, and convergent validity with other commonly used scales [52], and has been widely used in clinical and non-clinical populations, being validated also in transgender youths [63]. The scale is a self-report for use in patients suspected of ARFID, children or adults, but a version for parents has also been formulated [64], showing similar psychometric properties to the original version and allowing for convergence between children and adolescents and their parents to be assessed [65]. However, the scale does not distinguish ARFID well from other eating disorders, since patients with other eating disorders score high on the NIAS as well [66,67]. The fact is that the discriminatory point, i.e., the absence of shape/weight concerns, is not always present in ARFID patients, who may exhibit some comorbidity with other eating disorders [1]. For these reasons, the tool is employed conjointly with other eating disorder questionnaires and interviews.

Another scale recently introduced is the Pica, ARFID and Rumination disorder interview ARFID questionnaire (PARDI-AR-Q) [68]. This is a structured interview that distinguishes between these three clinical entities. It showed good psychometric properties, with internal consistency and discriminant and convergent validity and identified three factors with an exploratory factor analysis that each correspond to the already identified ARFID phenotypes, i.e., food avoidance on the basis of the food’s sensory characteristics, lack of interest in food or eating, and worry about aversive eating/food consequences [69,70]. The PARDI showed higher positive predictive values than the NIAS [71]. On the other hand, the NIAS was better at pinpointing the symptoms of ARFID [71]. However, the tool needs replication in large and diverse samples, since it compared adolescents and young adults with healthy controls in small samples (*n* = 113 for Bryant-Waugh et al., 2022 [69] and *n* = 163 for Cooper-Vince et al., 2022 [70]).

In summary, it is better to use both self-rated and clinician-rated tools for diagnosing ARFID, although this cannot address the persisting questions about ARFID [72]. The DSM-5-TR reworded the first ARFID criterion, deleting “as manifested by persistent failure to meet appropriate nutritional and/or energy needs” so as to better suit clinical needs, but it is felt that further improvement in DSM criteria is needed to address the above questions [72].

### 4.6. Eating Disturbances Across the Psychosis Spectrum

Disturbed eating behaviours are increasingly recognised as clinically relevant features within the schizophrenia spectrum, extending beyond the effects of antipsychotic treatment or metabolic dysregulation. Early conceptual work highlighted that restrictive eating, bingeing, purging, and food refusal may occur across different phases of schizophrenia, often embedded within broader disturbances of thought, affect, and self-experience [73]. More recent syntheses have confirmed that altered eating patterns in psychosis are highly heterogeneous and may reflect multiple underlying mechanisms, including negative symptoms, obsessive–compulsive traits, sensory hypersensitivity, delusional beliefs about food, and impaired interoceptive awareness [74]. Importantly, emerging evidence suggests that eating-related psychopathology in psychosis is not merely comorbid, but may be meaningfully linked to core symptom features and phenotype presentations of the disorder. In particular, disturbances of the basic sense of self, characterised by diminished self-presence, bodily alienation, and anomalous self-monitoring, have been shown to correlate with eating disorder symptomatology in individuals with early psychosis [75]. This association supports the hypothesis that disordered eating may represent a behavioural expression of deeper disruptions in embodiment and self-integration, rather than an isolated secondary condition. Taken together, these findings point to a transdiagnostic interface in which eating disturbances and psychotic vulnerability converge around alterations in bodily experience, self-boundaries, and affect regulation, underscoring the need for integrated conceptual and clinical models. From a clinical perspective, these observations highlight the importance of systematically assessing eating behaviours and dietary patterns in individuals with psychosis or psychosis-spectrum vulnerability, even in the absence of overt eating disorder diagnoses.

### 4.7. Pharmacological Considerations and Treatment Challenges

The pharmacological management of ARFID remains challenging due to the absence of disorder-specific medications and the reliance on off-label treatments targeting associated symptoms rather than core mechanisms. Neurobiological models conceptualise ARFID as a multidimensional condition involving sensory processing, anxiety regulation, and reward circuitry, none of which are directly addressed by current pharmacological options [76,77]. In cases where ARFID co-occurs with psychotic disorders, antipsychotic treatment may be necessary to manage psychotic symptoms; however, its impact on avoidant or restrictive eating behaviours is often limited, if not worsening them. Moreover, antipsychotic-induced metabolic syndrome, including weight gain, insulin resistance, and dyslipidaemia, poses additional clinical challenges, potentially exacerbating distress related to food, body sensations, and metabolic changes in this population [78]. Taken together, these factors highlight a structural gap between available pharmacological strategies and the embodied, sensory-driven features of ARFID, underscoring the need for integrated treatment approaches that combine pharmacotherapy with targeted nutritional and psychotherapeutic interventions.

### 4.8. Psychotherapeutic and Nutritional Interventions: Opportunities and Constraints

Psychotherapeutic and nutritional interventions represent potentially valuable adjunctive strategies in psychosis, particularly when restrictive or avoidant eating patterns such as ARFID are present. Nutritional psychiatry frameworks suggest that dietary interventions may improve metabolic health and quality of life in schizophrenia-spectrum disorders; however, evidence remains preliminary and limited by heterogeneous methodologies and uncertain effects on core psychopathology [79,80]. In patients with ARFID-like presentations, additional challenges arise from sensory sensitivity, avoidance, and low interoceptive awareness, which may limit engagement with standard nutritional approaches. Similarly, while psychotherapeutic group interventions can support emotional regulation and social functioning, adherence in psychosis is often compromised, particularly in individuals with negative symptoms or cognitive impairment [81]. These constraints highlight the need for individually tailored, developmentally informed interventions that explicitly address avoidance, embodiment, and sensory processing in order to enhance feasibility and therapeutic impact at the ARFID–psychosis interface.

The risk of bias of the pool of studies mentioned above proved to be low (Supplementary Table S2). However, the heterogeneity of the considered studies does not allow us to draw firm conclusions about their validity.

### 4.9. Limitations and Future Directions

The current evidence base is constrained by the paucity of systematic studies, reliance on isolated case reports, and marked heterogeneity of clinical presentations. The available data do not support conceptualising ARFID and psychosis as expressions of a shared diagnostic entity. Rather, they are more consistent with a model of shared vulnerability architecture involving sensory processing anomalies, interoceptive dysregulation, cognitive rigidity, and disturbances in self-integration. Furthermore, the discrepancy between empirical, data-based evidence and theories about the pathogenesis of ARFID reflects the novelty of the diagnostic category. The available evidence does not resolve the question of whether ARFID and psychotic conditions coexist because the former triggers psychosis or because the latter promotes the ARFID-type eating disorder. Moreover, despite favourable risk-of-bias reports (Supplementary Table S2), the Cochrane tools we used [82,83] are not particularly suitable for the assessment of the studies we commented on here. Finally, the mere fact that we conducted a non-systematic narrative review instead of a systematic one may have affected the obtained results and may have diverted the interpretation of the conclusions.

Future research should prioritise longitudinal and developmentally informed studies integrating neurodevelopmental, interoceptive, and computational approaches to clarify mechanisms underlying this overlap. Such work may improve diagnostic precision and inform more targeted and integrated clinical interventions for individuals presenting at this complex and under-recognised interface. Furthermore, studies should focus on strategies to deal with ARFID or to prevent it, such as harm reduction models of care with brief medical admissions when required so as to achieve emergency medical stabilisation or the utilisation of early intervention services to address prodromal symptoms [84]. Other studies should assess the diagnostic boundaries between ARFID and psychosis-related food refusal; to date, ARFID has been compared only with anorexia nervosa and identified food neophobia as a distinguishing factor [85]. A focus should be placed on the neurodevelopmental underpinnings of ARFID [86] and on the pathophysiological mechanisms. Regarding the latter, endocrine levels and responses were found to differ between persons with ARFID and healthy controls regarding PYY [87,88], ghrelin [88], and oxytocin [89]. This last finding offers support for a neurodevelopmental mechanism. Finally, as to the possibility of a pharmacological treatment of ARFID, this has been tested in animals [90] and barely in humans (one case has been successfully treated with the noradrenaline/serotonin receptor-specific antagonist antidepressant mirtazapine) [91]. Given the age of those involved, this is fair.

## 5. Conclusions

This narrative review suggests that ARFID and psychosis, while diagnostically distinct, may intersect in a subset of individuals through partially overlapping vulnerability dimensions rather than shared disease mechanisms. Sensory hypersensitivity, avoidance-based behaviours, interoceptive dysregulation, and disturbances in self-integration appear to represent potential points of convergence that can shape clinical presentation and complicate differential diagnosis.

The available evidence remains limited and largely based on observational data and isolated case reports, preventing us from drawing firm conclusions regarding prevalence or causality. Nonetheless, recognising these overlapping vulnerabilities has important clinical implications, as misattributing restrictive eating to either ARFID or psychosis alone may delay appropriate intervention and increase medical risk. Future research should adopt developmentally informed and transdiagnostic approaches to clarify underlying mechanisms and support more precise and integrated clinical management.

## Figures and Tables

**Table 1:** Studies and reviews focusing on the prevalence of avoidant/restrictive food intake disorder (ARFID) in various populations in chronological order.

Study	Prevalence Rates	Population
Murray et al., 2020 [24] ^#^i	6.3% clinical, 17.3% subclinical	Retrospective review chart of 410 consecutive patients at a tertiary gastroenterology service, aged 18–90 years; 73.0% female. 97 cases, 26 clinical ARFID, 71 subclinical; 92.8% (*n* = 90) motivated eating avoidance/restriction with fear of gastrointestinal symptoms
Kambanis et al., 2020 [2]	100% *	ARFID (*n* = 62) or subthreshold ARFID (*n* = 12), total *n* = 74 (38 males, 36 females; 65 adolescents, 9 adults, mean age 15.0 years, SD = 3.5); 45% with current psychiatric comorbidity, 53% with lifetime psychiatric comorbidity; 8% currently suicidal, 14% lifetime suicidal
Bertrand et al., 2021 [25]	3.0%	Interview with 401 French families for ARFID symptoms or feeding and eating disorders (excluding anorexia nervosa, bulimia nervosa, binge eating disorder, other specified feeding and eating disorder, pica or rumination) in their children (age range 0–18 years); the prevalence of unspecified feeding and eating disorder was 9.7%
Koomar et al., 2021 [26]	21% of probands at high risk	US cohort of 5157 probands with autism; up to 17% of parents of children with autism are at heightened risk for ARFID (narrow-sense heritability for ARFID risk = 0.45)
D’Adamo et al., 2023 [27]	4.7%	50,082 adult US respondents to a survey (general population); 2378 (4.7%) adult respondents screened positive for ARFID (80% lack of interest in eating, 55.4% food sensory avoidance, and 30.8% fear of negative food consequences)
Medina-Tepal et al., 2023 [28]	5–22%	Systematic review of 47 case studies
Sader et al., 2023 [29]	6.4%	2862 Dutch children (49.2% male); 183 (54.6% male) screened positive for ARFID
Sanchez-Ceredo et al., 2023 [23]	0.3–15.5% non-clinical; specialist outpatient 32–64%; specialised 5–22%	Review of 30 studies. Psychiatric comorbidity: anxiety disorders, 9.1–72%; autism spectrum disorder, 8.2–54.75%. Data from various services and using different assessment methods. Specialised eating disorder services, specialist feeding clinics, non-clinical samples and national surveillance
Weeks et al., 2023 [30]	13–40%	Review; focus on disorders of the gut–brain interaction. Higher prevalence rates reported by patient surveys for both adults and children than by retrospective chart reviews. Children with more definite ARFID diagnoses than adults in outpatient gastroenterology services (8% vs. 6%), but with less ARFID-related symptoms (15% vs. 17%). Cases identified by surveys ranged from 19% to 40%
Van Buuren et al., 2023 [31]	1.98%	Survey of 5072 adolescents from the general New South Wales (Australia) population; girls outnumber boys by a little, with a weak effect size. Distress and quality of life did not differ between ARFID and non-ARFID groups
Burton-Murray et al., 2024 [32] ^#^	11%	101 US adults with ulcerative colitis in remission (mean age 49.9 years, SD 16.5; 55% women); about 30% of patients were positive for other feeding and eating disorders
Almeida et al., 2024 [33]	7.56%	344 US patients with a feeding and eating disorder consulting a gastroenterology service during 2010–2020 (ARFID was introduced in the DSM-5 in 2013); most were diagnosed with a feeding and eating disorder already at gastroenterology consultation (NN = 255, 74.2%), 82 (23.8%) were diagnosed with a feeding and eating disorder after consultation; of them, 59 were diagnosed as eating disorder not otherwise specified, and of the latter, 7 had ARFID (11.86%). 84.6% of the 26 patients meeting DSM-5 ARFID criteria had a functional/motility GI disorder
Nicholls-Clow et al., 2024 [22]	11.4%	Systematic review of 26 studies. Random effects meta-analysis. Quality effects reduced this figure to 4.5%
Menzel and Perry, 2024 [34]	0.3–15–5% non-clinical; 5–64% non-clinical	General review. Epidemiology reported for clinical and non-clinical samples
Martin et al., 2025 [35]	33% strict, 49% lenient criteria	Retrospective chart review of 33 UK patients with gut–brain interaction disorders (median age 44.3, SD = 15.5, range 18–73 years, 29 women [88%]). Assessed for ARFID according to strict vs. lenient criteria. Only 18% of patients did not meet criteria for ARFID; 11 were diagnosed as ARFID by strict criteria and 16 met lenient criteria
Matherne et al., 2025 [36]	42% patients; 55% caregivers	38 US adolescents with disorders of brain–gut interaction (mean age 14.74 years, SD = 1.69; age range 12–17 years; 71% girls). ARFID symptoms reported by patients and their primary caregiver; patient and caregiver reports showed high internal consistency
Mikhael-Moussa et al., 2025 [37]	10–80%	Scoping review of 18 studies reporting data of gastroenterology clinics; prevalence of ARFID symptoms in neurogastroenterology patients 10–80%, prevalence of neurogastroenterology disorders in ARFID patients 7–60%
Rezaei et al., 2025 [38]	13.2–40%	Scoping review of 9 studies reporting on ARFID in patients with disorders of gut–brain interaction and vice versa; ARFID patients show gastrointestinal symptoms in the range of 7–100%
Dinkler et al., 2025 [39]	6.5%	Parents of 645 children (50.5% male, mean age 3.2 years) completed a screen for ARFID at 2.5- and 4-year routine check-ups at 21 child health centres in West Sweden; 42 screened positive, of whom 21 received ARFID diagnosis. Early language delays in 39.1% of children with ARFID vs. 13.5% without ARFID
Abber et al., 2025 [40]	100% *	159 ARFID children and adolescents referring to a US multisite eating disorder treatment centre (aged 9–18, 63% female); latent profile analysis identified 4 ARFID prototypes: 1, fear of aversive consequences (*n* = 26); 2, sensory-based avoidance and lack of interest (*n* = 43); 3, with all prototypes (*n* = 44); and 4, Non-Endorsers (scoring low on questionnaires, *n* = 53)
Brownlow et al., 2025 [41]	26%	4002 people from the adult general population of UK and US (mean age = 47.1 years, 50% women) completed a survey. Higher in women (29.6%) than men (22.1%) and varied according to the age range: 18–39 years, 31.6%; 40–64 years, 25.0%; ≥65 years, 16.1%
Hog and Dinkler, 2025 [42]	2.84% non-clinical; 12.0% clinical	Review of recent studies (2021–2023 selected, 2024–2025 all) reporting prevalence in clinical and nonclinical populations
Califano et al., 2025 [43]	6.9%	Retrospective chart review of 72 Italian children and adolescents with feeding and eating disorders. All participants had psychiatric comorbidities: 66.5% with mood disorders, 87.5% with anxiety disorders, 47.2% with obsessive–compulsive and related disorders, 30.5% with attention-deficit/hyperactivity disorder, 13.9% with disruptive and impulse-control disorders, and 40.3% with psychotic symptoms
Novo et al., 2025 [44]	3.1%	Portuguese children; (*n* = 5; 4 [80% male]; mean age = 5.8 years, SD = 2.17)
Flack et al., 2025 [45]	26%	Same population of Brownlow et al. (2025) [41], different set of data provided. Prevalence of ARFID in people with disorders of gut–brain interaction than in those without (34.6% vs. 19.4%). Data were similar for the UK and the US. Motives for ARFID were lack of interest in eating (21.5%), sensory-based avoidance (18.1%), and fear of aversive consequences of food (9.9%)
Kim et al., 2025 [5]	17.35%	392 Korean outpatients with ARFID (*n* = 68) or restrictive anorexia nervosa (*n* = 324) at an eating disorders clinic; the disorders were clinically distinguishable
Islamoğlu et al., 2025 [46]	Not calculated	25 Turkish children with autism spectrum disorder vs. 30 typically developing; mean age = 8.02 years; SD = 3.28; 61.8% male; children with autism scored significantly higher on the ARFID scale
Kramer et al., 2026 [47]	75.5% children and 64.4% of those undergoing diagnostic interview	Online survey of parents of children from the community (Germany) aged 2–17 years, 60.4% male vs. adults aged 18–73 years, 76.8% female. Analysable data from 270 parents of children and 491 adults. 98 parents and 149 adults underwent diagnostic interview

* ARFID-only sample. ^#^ The senior author is the same. SD, standard deviation.

## Data Availability

The review used published data; hence, no new data were created. All data are contained in the manuscript and are accessible to all.

## Supplementary Materials

The following supporting information can be downloaded at https://www.mdpi.com/article/10.3390/jcm15051704/s1, Supplementary Table S1. Detailed search strategy for each of the investigated databases; Supplementary Table S2. Cochrane tool for assessing risk of bias (RoB-2 and ROBINS-I v.2).
