# Supplementary material for: Co-Occurrence of Avoidant/Restrictive Food Intake Disorder (ARFID) and Schizophrenia-Spectrum Disorders: A Comprehensive Review
Source: J Clin Med. 2026 Feb 24;15(5):1704. doi: 10.3390/jcm15051704 (PMC12986347; doi:10.3390/jcm15051704)
Supplement: Supplementary file 1 [file jcm-15-01704-s001.zip › jcm-4156811-supplementary.pdf]

Supplementary Material

Supplementary Table S1. Detailed search strategy for each of the investigated databases.

(ARFID OR Avoidant/restrictive food intake disorder) AND (psychosis OR psychotic OR schizophren* OR schizoaffect* OR mania OR manic OR bipolar OR schizotyp* OR schizoid OR paranoi*) 14.1.2026 **PubMed**→12 articles

N°	Article	Label
1	de la Peña FR, Rosetti MF, Rodríguez-Delgado A, Villavicencio LR, Palacio JD, Montiel C, Mayer PA, Félix FJ, Larraguibel M, Viola L, Ortiz S, Fernández S, Jaímes A, Feria M, Sosa L, Palacios-Cruz L, Ulloa RE. Construct validity and parent-child agreement of the six new or modified disorders included in the Spanish version of the Kiddie Schedule for Affective Disorders and Schizophrenia present and Lifetime Version DSM-5 (K-SADS-PL-5). J Psychiatr Res. 2018;101:28-33. doi: 10.1016/j.jpsychires.2018.02.029. Epub 2018 Mar 1.	Psychometrics
2	Wassenaar E, O'Melia AM, Mehler PS. A causality dilemma: ARFID, malnutrition, psychosis, and hypomagnesemia. Int J Eat Disord. 2018;51(9):1113-1116. doi: 10.1002/eat.22939. Epub 2018 Sep 7.	Case
3	Westfall NC, Mavrides NA, Coffey BJ. Multidisciplinary management of adolescent early-onset, treatment-resistant schizophrenia complicated by avoidant/restrictive food intake disorder and catatonia in acute exacerbations. J Child Adolesc Psychopharmacol. 2018;28(9):663-666. doi: 10.1089/cap.2018.29157.bjc. Epub 2018 Nov 14.	Case
4	Chen YL, Chen WJ, Lin KC, Shen LJ, Gau SS. Prevalence of DSM-5 mental disorders in a nationally representative sample of children in Taiwan: methodology and main findings. Epidemiol Psychiatr Sci. 2019;29:e15. doi: 10.1017/S2045796018000793.	Epidemiological
5	Kambanis PE, Kuhnle MC, Wons OB, Jo JH, Keshishian AC, Hauser K, Becker KR, Franko DL, Misra M, Micali N, Lawson EA, Eddy KT, Thomas JJ. Prevalence and correlates of psychiatric comorbidities in children and adolescents with full and subthreshold avoidant/restrictive food intake disorder. Int J Eat Disord. 2020;53(2):256-265. doi: 10.1002/eat.23191. Epub 2019 Nov 8.	Epidemiological
6	Kambanis PE, Harshman SG, Kuhnle MC, Kahn DL, Dreier MJ, Hauser K, Slattery M, Becker KR, Breithaupt L, Misra M, Micali N, Lawson EA, Eddy KT, Thomas JJ. Differential comorbidity profiles in avoidant/restrictive food intake disorder and anorexia nervosa: Does age play a role? Int J Eat Disord. 2022;55(10):1397-1403. doi: 10.1002/eat.23777. Epub 2022 Jul 18.	Opinion
7	Fekih-Romdhane F, Hallit R, Malaeb D, Sakr F, Dabbous M, Sawma T, Obeid S, Hallit S. Psychometric properties of an Arabic translation of the Nine Item Avoidant/Restrictive Food Intake Disorder Screen (NIAS) in a community sample of adults. J Eat Disord. 2023;11(1):143. doi: 10.1186/s40337-023-00874-0.	Psychometrics
8	Salameh G, El Khoury N, Hallit R, Malaeb D, Sakr F, Dabbous M, Fekih-Romdhane F, Obeid S, Hallit S. The mediating effect of dysmorphic concern in the association between avoidant restrictive food intake disorder and suicidal ideation in adults. BMC Psychiatry. 2024;24(1):42. doi: 10.1186/s12888-023-05490-5.	Survey
9	Chaaya R, Hallit R, Malaeb D, Sakr F, Dabbous M, El Khatib S, Fekih-Romdhane F, Hallit S, Obeid S. Moderating effect of self-esteem between perfectionism and avoidant restrictive food intake disorder among Lebanese adults. BMC Psychiatry. 2024;24(1):325. doi: 10.1186/s12888-024-05762-8.	No psychosis
10	Li R, Radhakrishnan V. A case of avoidant / restrictive food intake disorder in an adult with schizophrenia and obsessive-compulsive disorder. J Acad Consult Liaison Psychiatry. 2025;66(3):268-269. doi: 10.1016/j.jaclp.2025.01.005. Epub 2025 Jan 30.	Case
11	Califano M, Pruccoli J, Cavallino O, Lenzi A, Parmeggiani A. Psychopathological comorbidities in children and adolescents with feeding and eating disorders: An Italian clinical study. Pediatr Rep. 2025;17(3):61. doi: 10.3390/pediatric17030061.	Epidemiological
12	Chammas G, Hallit S, Abou Nader L, Chammas M, Fekih-Romdhane F, Obeid S, Haddad G. Mediating effect of food disgust between depression/anxiety and avoidant restrictive eating. J Eat Disord. 2025;13(1):244. doi: 10.1186/s40337-025-01430-8.	No psychosis
13	Kopańska M, Łucka I, Siegel M, Trojniak J, Pąchalska M. From ARFID to binge eating: A review of the sensory, behavioral, and gut-brain axis mechanisms driving co-occurring eating disorders in children and adolescents with autism spectrum disorder. Nutrients. 2025;17(23):3714. doi: 10.3390/nu17233714.	No psychosis
14	Abou Nader L, Chammas G, Chammas M, Fekih-Romdhane F, Hallit S, Obeid S. The mediating effect of alexithymia between Avoidant/Restrictive Food Intake Disorder (ARFID) and suicidal ideation among a sample of Lebanese adults. PLoS One. 2026;21(1):e0340095. doi: 10.1371/journal.pone.0340095.	No psychosis

(TITLE (ARFID OR Avoidant / restrictive food intake disorder) AND TITLE-ABS-KEY (psychosis OR psychotic OR schizophrenia OR schizophrenic OR schizoaffective OR mania OR manic OR bipolar OR schizotypal OR schizoid OR paranoid OR paranoia)) **Scopus** 14.2.2026 → 11 results

N°	Article	Label
1	Okereke NK. Buspirone treatment of anxiety in an adolescent female with avoidant/restrictive food intake disorder. J Child Adolesc Psychopharmacol. 2018;28(6):425-426. doi: 10.1089/cap.2018.0005. Epub 2018 May 29.	Case
2	Westfall NC, Mavrides NA, Coffey BJ. Multidisciplinary management of adolescent early-onset, treatment-resistant schizophrenia complicated by avoidant/restrictive food intake disorder and catatonia in acute exacerbations. J Child Adolesc Psychopharmacol. 2018;28(9):663-666. doi: 10.1089/cap.2018.29157.bjc. Epub 2018 Nov 14.	Dupl 3PM
3	Kambanis PE, Kuhnle MC, Wons OB, Jo JH, Keshishian AC, Hauser K, Becker KR, Franko DL, Misra M, Micali N, Lawson EA, Eddy KT, Thomas JJ. Prevalence and correlates of psychiatric comorbidities in children and adolescents with full and subthreshold avoidant/restrictive food intake disorder. Int J Eat Disord. 2020;53(2):256-265. doi: 10.1002/eat.23191. Epub 2019 Nov 8.	Dupl 5PM

4	Kambanis PE, Harshman SG, Kuhnle MC, Kahn DL, Dreier MJ, Hauser K, Slattery M, Becker KR, Breithaupt L, Misra M, Micali N, Lawson EA, Eddy KT, Thomas JJ. Differential comorbidity profiles in avoidant/restrictive food intake disorder and anorexia nervosa: Does age play a role? Int J Eat Disord. 2022;55(10):1397-1403. doi: 10.1002/eat.23777. Epub 2022 Jul 18.	Dupl 6PM
5	Chovel Sella A, Hadaway N, Stern C, Becker KR, Holsen LM, Eddy KT, Micali N, Misra M, Thomas JJ, Lawson EA. Lower ghrelin levels are associated with higher anxiety symptoms in adolescents and young adults with avoidant/restrictive food intake disorder. J Clin Psychiatry. 2023;84(3):22m14482. doi: 10.4088/JCP.22m14482.	No psychosis
6	Aulinas A, Muhammed M, Becker KR, Asanza E, Hauser K, Stern C, Gydus J, Holmes T, Murray HB, Breithaupt L, Micali N, Misra M, Eddy KT, Thomas JJ, Lawson EA. Oxytocin response to food intake in avoidant/restrictive food intake disorder. Eur J Endocrinol. 2023;189(2):149-155. doi: 10.1093/ejendo/lvad087.	No psychosis
7	Passarini S, Guerrera S, Picilli M, Fucà E, Casula L, Menghini D, Pirchio S, Zanna V, Valeri G, Vicari S. The challenge of a late diagnosis of Autism Spectrum Disorder: co-occurring trajectories and camouflage tendencies. a case report of a young Autistic female with Avoidant Restrictive Food Intake Disorder. Front Psychiatry. 2025;15:1447562. doi: 10.3389/fpsyt.2024.1447562.	Case
8	Bennett N, Matthews A. Diagnostic uncertainty: Avoidant/restrictive food intake disorder and co-occurring psychosis in a severely malnourished adolescent male. Psychiatry Res Case Rep. 2024;3(1):100197. doi: 10.1016/j.psycr.2023.100197.	Case
9	Rozzell-Voss KN, Becker KR, Tabri N, Dreier MJ, Wang SB, Kuhnle M, Gydus J, Burton-Murray H, Breithaupt L, Plessow F, Franko D, Hauser K, Asanza E, Misra M, Eddy KT, Holsen L, Micali N, Thomas JJ, Lawson EA. Trajectory of ghrelin and PYY around a test meal in males and females with avoidant/restrictive food intake disorder versus healthy controls. Psychoneuroendocrinology. 2024;167:107063. doi: 10.1016/j.psyneuen.2024.107063. Epub 2024 May 6.	No psychosis
10	Presseller EK, Cooper GE, Thornton LM, Birgegård A, Abbaspour A, Bulik CM, Forsén Mantilla E, Dinkler L. Assessing avoidant/restrictive food intake disorder (ARFID) symptoms using the nine item ARFID Screen in >9000 Swedish adults with and without eating disorders. Int J Eat Disord. 2024;57(11):2143-2155. doi: 10.1002/eat.24274. Epub 2024 Aug 8.	Psychometrics
11	Li R, Radhakrishnan V. A case of avoidant / restrictive food intake disorder in an adult with schizophrenia and obsessive-compulsive disorder. J Acad Consult Liaison Psychiatry. 2025;66(3):268-269. doi: 10.1016/j.jaclp.2025.01.005. Epub 2025 Jan 30.	Dupl 10PM

title(ARFID OR Avoidant/restrictive food intake disorder) AND summary(psychosis OR psychotic OR schizophren* OR schizoaffect* OR mania OR manic OR bipolar OR schizotyp* OR schizoid OR paranoi*) **PsycINFO/PsycARTICLES** 14.2.2026 → 5 results

**N°**	**Article**	**Label**
1	Westfall NC, Mavrides NA, Coffey BJ. Multidisciplinary management of adolescent early-onset, treatment-resistant schizophrenia complicated by avoidant/restrictive food intake disorder and catatonia in acute exacerbations. J Child Adolesc Psychopharmacol. 2018;28(9):663-666. doi: 10.1089/cap.2018.29157.bjc. Epub 2018 Nov 14.	Dupl 3PM 2Sc
2	Wassenaar E, O'Melia AM, Mehler PS. A causality dilemma: ARFID, malnutrition, psychosis, and hypomagnesemia. Int J Eat Disord. 2018;51(9):1113-1116. doi: 10.1002/eat.22939. Epub 2018 Sep 7.	Dupl 2PM
3	Kambanis PE, Kuhnle MC, Wons OB, Jo JH, Keshishian AC, Hauser K, Becker KR, Franko DL, Misra M, Micali N, Lawson EA, Eddy KT, Thomas JJ. Prevalence and correlates of psychiatric comorbidities in children and adolescents with full and subthreshold avoidant/restrictive food intake disorder. Int J Eat Disord. 2020;53(2):256-265. doi: 10.1002/eat.23191. Epub 2019 Nov 8.	Dupl 5PM 3Sc
4	Bennett N, Matthews A. Diagnostic uncertainty: Avoidant/restrictive food intake disorder and co-occurring psychosis in a severely malnourished adolescent male. Psychiatry Res Case Rep. 2024;3(1):100197. doi: 10.1016/j.psycr.2023.100197.	Dupl 8Sc
5	Li R, Radhakrishnan V. A case of avoidant / restrictive food intake disorder in an adult with schizophrenia and obsessive-compulsive disorder. J Acad Consult Liaison Psychiatry. 2025;66(3):268-269. doi: 10.1016/j.jaclp.2025.01.005. Epub 2025 Jan 30.	Dupl 10PM 11Sc

Supplementary Table S2. Risk of Bias assessment using ROBINS-I V2 tool

Study	Confounding	Classifications of interventions	Participant selection	Missing data	Measurement of outcome	Selection of reported results	Overall Risk of Bias
Murray et al., 2020 [24]	Low	Low	Low	Moderate	Low	Low	Moderate
Kambanis et al., 2020 [2]	Low	Low	Moderate	Low	Low	Low	Moderate
Bertrand et al., 2021 [25]	Low	Low	Moderate	Low	Moderate	Low	Moderate
Koomar et al., 2021 [26]	Low	Moderate	Moderate	Low	Moderate	Low	Moderate
D’Adamo et al., 2023 [27]	Low	Moderate	Moderate	Low	Moderate	Low	Moderate
Medina-Tepal et al., 2023 [28]	NA	NA	NA	NA	NA	NA	NA
Sader et al., 2023 [29]	Low	Low	Moderate	Moderate	Moderate	Low	Moderate
Sanchez-Ceredo et al., 2023 [23]	NA	NA	NA	NA	NA	NA	NA
Weeks et al., 2023 [30]	NA	NA	NA	NA	NA	NA	NA
Van Buuren et al,. 2023 [31]	Low	Low	Moderate	Moderate	Low	Low	Moderate
Burton-Murray et al., 2024 [32]	Low	Low	Low	Low	Moderate	Low	Moderate
Almeida et al., 2024 [33]	Low	Low	Low	Moderate	Moderate	Low	Moderate
Nicholls-Clow et al., 2024 [22]	NA	NA	NA	NA	NA	NA	NA
Menzel and Perry, 2024 [34]	NA	NA	NA	NA	NA	NA	NA
Martin et al., 2025 [35]	Low	Low	Moderate	Moderate	Moderate	Low	Moderate
Matherne et al., 2025 [36]	Low	Low	Moderate	Low	Moderate	Low	Moderate
Mikhael-Moussa et al., 2025 [37]	NA	NA	NA	NA	NA	NA	NA
Rezaei et al., 2025 [38]	NA	NA	NA	NA	NA	NA	NA
Dinkler et al., 2025 [39]	Low	Low	Moderate	Low	Low	Low	Moderate
Abber et al., 2025 [40]	Low	Low	Moderate	Moderate	Moderate	Low	Moderate
Brownlow et al., 2025 [41]	Low	Moderate	Moderate	Low	Moderate	Low	Moderate
Hog and Dinkler, 2025 [42]	NA	NA	NA	NA	NA	NA	NA
Califano et al., 2025 [43]	Low	Low	Low	Low	Low	Low	Low
Novo et al., 2025 [44]	Moderate	Low	Serious	Serious	Moderate	Moderate	Serious
Flack et al., 2025 [45]	Low	Low	Moderate	Low	Moderate	Low	Moderate
Kim et al., 2025 [5]	Moderate	Low	Low	Low	Low	Low	Moderate
Islamoğlu et al., 2025 [46]	Low	Moderate	Low	Low	Moderate	Low	Moderate
Kramer et al., 2026 [47]	Low	Low	Moderate	Low	Low	Low	Moderate

Low risk	Low risk<br>except for concerns about uncontrolled confounding (only for D1)	Moderate risk	Serious risk	Critical risk

Abbreviations: NA, Not applicable (received by review articles).

References

[82] Sterne, J.A.C.; Savović, J.; Page, M.J.; Elbers, R.G.; Blencowe, N.S.; Boutron, I.; Cates, C.J.; Cheng, H.-Y.; Corbett, M.S.; Eldridge, S.M.; Hernán, M.A.; Hopewell, S.; Hróbjartsson, A.; Junqueira, D.R.; Jüni, P.; Kirkham, J.J.; Lasserson, T.; Li, T.; McAleenan, A.; Reeves, B.C.; Shepperd, S.; Shrier, I.; Stewart, L.A.; Tilling, K.; White, I.R.; Whiting, P.F.; Higgins, J.P.T. RoB 2: a revised tool for assessing risk of bias in randomised trials. *BMJ* **2019**, *366*, l4898.

[83] Sterne J, Brandt Mathur M, Elbers R, Hróbjartsson A, McAleenan A, Reeves B, Shrier I, Tilling K, Armstrong R, Berkman N, Boutron I, Carpenter J, Chan A-W, Deeks J, Golder S, Henry D, Jüni P, Kirkham J, Konstantinidis M, Lasserson T, Loke Y, McGuinness L, Page M, Savović J, Shea B, Loke Y, Mawdsley D, Shepperd S, Tugwell P, Valentine J, Viswanathan M, Sharma Waddington H, Wells G, Hernán M, Higgins J. nThe Risk Of Bias In Non-randomized Studies – of Interventions, Version 2 (ROBINS-I V2) assessment tool (for follow-up studies) 20 November 2025. London (UK): Cochrane. Available from https://sites.google.com/site/riskofbiastool/welcome/robins-i-v2, assessed on December 19, 2025.
